# Enhancement Patterns of Gastric Carcinoma on Contrast-Enhanced Ultrasonography: Relationship with Clinicopathological Features

**DOI:** 10.1371/journal.pone.0073050

**Published:** 2013-09-06

**Authors:** Fang Wei, Pintong Huang, Shiyan Li, Jian Chen, Ying Zhang, Yurong Hong, Shumei Wei, David Cosgrove

**Affiliations:** 1 Department of Ultrasound, The 2nd Affiliated Hospital of Zhejiang University College of Medicine, Hangzhou, China; 2 Department of Diagnostic Ultrasound and Echocardiography, Sir Run Run Shaw Hospital, Zhejiang University College of Medicine, Hangzhou, China; 3 Department of Surgery, The 2nd Affiliated Hospital of Zhejiang University College of Medicine, Hangzhou, China; 4 Department of Pathology, The 2nd Affiliated Hospital of Zhejiang University College of Medicine, Hangzhou, China; 5 Imaging Sciences Department, Imperial College, Hammersmith Hospital, London, United Kingdom; Northwestern University Feinberg School of Medicine, United States of America

## Abstract

The aim of this study was to assess the relationship between the enhancement patterns and clinicopathological features of gastric cancer using intravenous contrast-enhanced ultrasonography (CEUS). In this Ethics Committee-approved prospective study, five hundred fifty two patients with gastric cancer who gave informed consent were examined preoperatively with CEUS. The enhancement pattern of each tumor was analyzed visually. Gross and histopathological findings on the postoperative specimens were compared with the preoperative CEUS findings. The most common CEUS pattern in differentiated gastric cancer was homogeneous enhancement, whereas heterogeneous enhancement was the most common pattern in undifferentiated gastric cancer. The proportion of heterogeneous enhancement was significantly different between the two histological subtypes (Chi- square = 146.735, *P*<0.001). The sensitivity and specificity of early heterogeneous enhancement on CEUS in diagnosing undifferentiated gastric cancer were 78.84% and 72.59% respectively. Gastric cancers with heterogeneous enhancement were more often Borrmann III and IV macroscopic types than those with homogeneous enhancement (66.56% vs. 30.80%, *P<*0.001), more commonly T3 and T4 depth of invasion than those with homogeneous enhancement (71.52% vs. 59.60%, *P<*0.05), more often showed lymphatic invasion than those with homogeneous enhancement (84.44% vs. 76.40%, *P<*0.05), and were less likely to receive curative gastrectomy than those with homogeneous enhancement (74.83% vs. 86.40%, *P*<0.005). The intra- and inter-observer reproducibility were both almost perfect for assessing enhancement patterns, with Kappa values of 0.916 (*P*<0.001) for intra-observer and 0.842 (*P*<0.001) for inter-observer reproducibility. CEUS provided detailed information about tumor vascularity and contrast enhancement patterns in gastric cancer. CEUS is promising as a new and useful method to predict the histological type of gastric cancer.

## Introduction

Gastric cancer is one of the most common malignancies worldwide and the prognosis remains poor [1], [2]. Complete resection is the best option for curative treatment, but less than half of patients are expected to survive for more than 5 years [3]. Accurate pretreatment assessment provides information on potential curability as well as for developing the optimal therapeutic strategy [4]. It has been shown that the clinicopathological characteristics of gastric cancer are linked with prognosis and recurrence according to the stage of disease, even in patients with the same clinicopathological stage. Preoperative recognition of the clinicopathological factors is indispensable for predicting the risk of recurrence and deciding the patient’s individualized postoperative surveillance schedule [5].

Conventionally, many modalities, such as computed tomography (CT) and endoscopy have been used for assessing gastric carcinoma. Multi-detector CT (MDCT) with multi-planar reformatted views is a powerful test for non-invasive evaluation of gastric cancer [6]. However, it carries a burden on ionizing radiation, which may be a disadvantage [7]. Upper digestive tract endoscopy is the gold standard for the diagnosis of gastric tumors and has improved the accuracy of diagnosis [8]. When combined with biopsy and brush cytology, endoscopy has an overall sensitivity of 95%–98% in the detection of gastric cancer [9], [10]. Unfortunately, endoscopy is not accepted by all patients, as it is rather invasive and traumatic. In addition, endoscopy has a limited role in diagnosing the linitis plastica type of gastric carcinoma in which sensitivity varies from 33 to 73 percent [11]–[14].

The use of transabdominal ultrasonography for the stomach wall is limited because of interference by intragastric gas. Oral contrast-enhanced ultrasound (OCEUS) improves imaging by displacing the air in the stomach and by distending the gastric lumen, thus helping to display mucosal lesions. Detection of gastric cancer can be improved by OCEUS [15], [16]. Furthermore, OCEUS combined with intravenous CEUS, improves the assessment of the microvessel density of gastric carcinomas [17]. Because the microbubbles of intravenous contrast materials flow with red blood cells and do not cross into the interstitial space [18], [19], they behave as a true intravascular marker and demonstrate the density of vascularization of tissues. Modern multipulse imaging methods, such as the contrast pulse sequencing (CPS) technology, effectively suppress tissue echoes, so that the microvasculature of a tumor can be detected [20], [21].

To our knowledge, there has been no report of the relationship between CEUS findings and the clinicopathological characteristics of gastric carcinomas. The purpose of our study was to assess the value of CEUS as a method to improve the preoperative assessment of the clinicopathological features of gastric carcinomas.

## Methods

### Subjects

The ethics committee of the second affiliated hospital of Zhejiang University College of Medicine approved this prospective study. Written informed consent was obtained from all patients prior to their examination. Between August 2009 and January 2013, 683 in-patients with adenocarcinomas of the stomach proven by endoscopic biopsy were scheduled for gastrectomy. All were assessed with CEUS preoperatively and were enrolled in this study subject to the following exclusion criteria: (i) patients (27 cases) previously treated with nonsteroidal anti-inflammatory drugs, chemotherapy, radiotherapy or immunotherapy; (ii) elderly patients with comorbidities for surgery (21 cases); (iii) unresectable lesions with metastases detected on preoperative evaluation (46 cases); (iv) missing data on CEUS, morphology or clinical pathology (13 cases); (v) lesions too small (thickness less than 3 mm) to be visualized on OCEUS (24 cases). The remaining 552 patients (217 females, 335 males, mean age 65±14 years) were included in the study. Surgical resections were performed within 5 days after the CEUS examination.

### Ultrasonography

CEUS was performed by either one of two trained radiologists (Y Z and Y H, with 12 and 10 years experience respectively). The patients fasted for at least 6 hours and atropine sulfate (0.05 mg/kg) was administered via intramuscular injection 30 minutes before the examination to inhibit gastric peristalsis. An Acuson Sequoia 512 system (Siemens, MountainView, CA, USA), equipped with a 4V1 vector™ transducer (frequency 1.0 to 4.0 MHz) and contrast pulse sequencing (CPS) technology was used [22]. In CPS three sequential pulses of differing phase and amplitude (positive full power, positive half power and negative full power) are transmitted along each acoustic line; when summed, the linear signals from tissue are suppressed, leaving the non-linear microbubble signals that are used to form the microbubble-specific image.

The oral contrast agent Xinzhang® (Huqingyutang, Hangzhou, China) was supplied as a powder composed of a soya derivative (48 g per package), as used in the previous study [8]; it was reconstituted by adding 500 mL of boiling water and gently agitating by hand to form a homogeneous suspension. After cooling to a room temperature, the patient was asked to drink the palatable liquid as quickly as possible. It dilates the stomach and displaces the air within it so that the lumen appears as a homogeneous mid-gray on B-mode imaging and provides an acoustic window that lasts for around 60 minutes. It does not appear on microbubble-specific images since it behaves like tissue, with minimal non-linear properties.

The OCEUS examination was performed in detail as previously described [17]. The two radiologists were aware of the diagnosis of gastric cancer prior to OCEUS examination but did not have any other clinicopathological information on the patients’ condition. The distal esophagus and the cardia were observed in real time whilst the patients ingested the oral agent. Then the remaining parts of the stomach were examined in turn with the patient in right lateral or supine positions. When the lesion was displayed clearly, its thickness was measured. The scanner’s zoom control was used to improve the spatial resolution, if necessary. After the OCEUS examination, dynamic real-time intravenous CEUS was performed following the injection of 2.4 mL of SonoVue (Bracco SpA, Milan, Italy) as a bolus via a 19-gauge peripheral venous cannula, followed by a 10 mL saline flush using the CPS mode at a frequency of 1.5 MHz and an acoustic power of −15 to −21 dB. This resulted in a low mechanical index (0.20), which minimized microbubble disruption. A timer on the sonographic unit was activated at the beginning of the injection, and the entire movie sequence (at least 5 minutes) was stored on magneto-optical disks for analysis.

### Imaging and Histological Analyses

**Figure 1:**
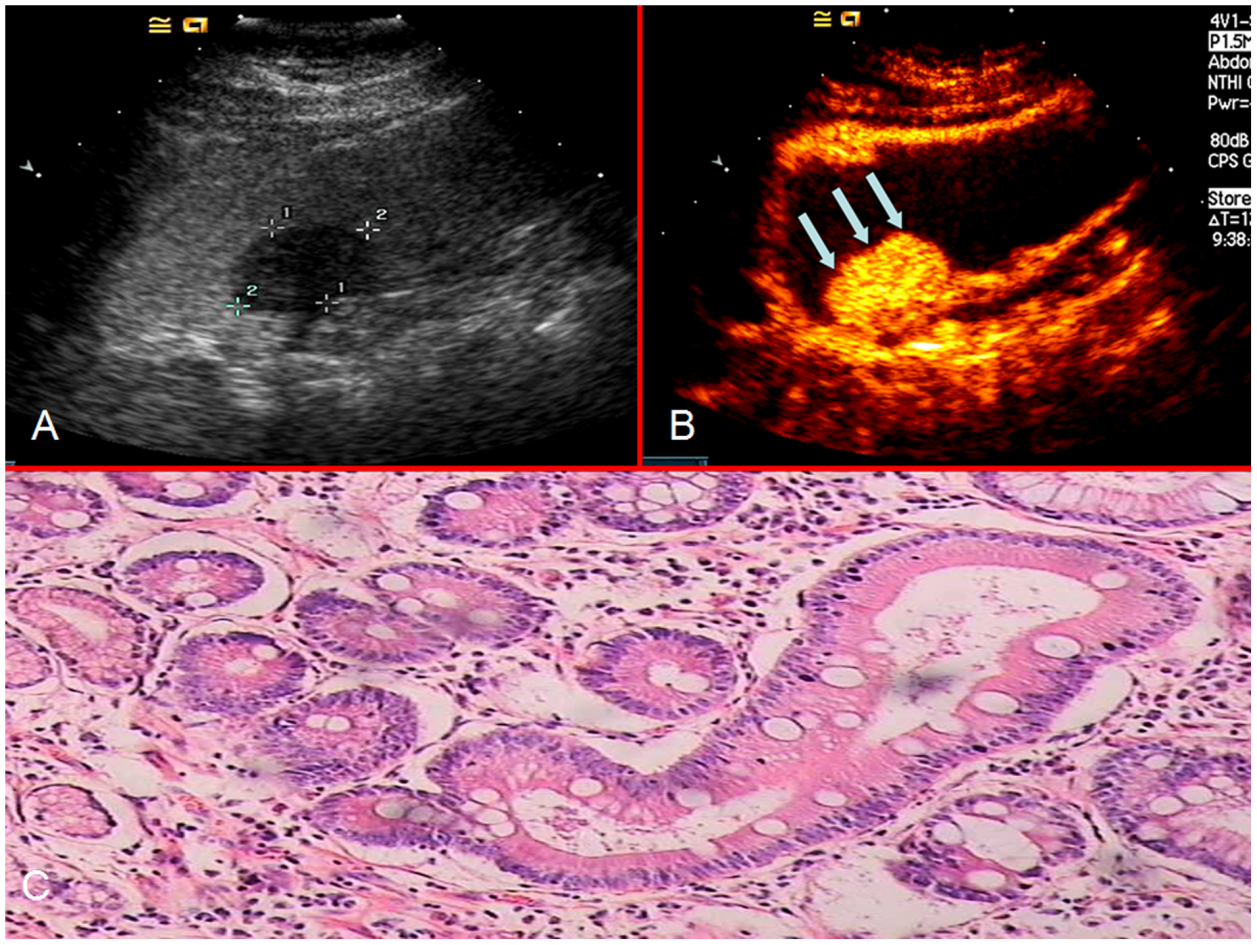
OCEUS (a) shows the thickened gastric wall with nodular polypoid appearance (arrow), while CEUS (b) showed that the thickened gastric wall (arrow in a) enhanced homogeneously during the arterial phase. Tubular adenocarcinoma with good differentiation was proven by histopathological examination (c) (x100).

**Figure 2:**
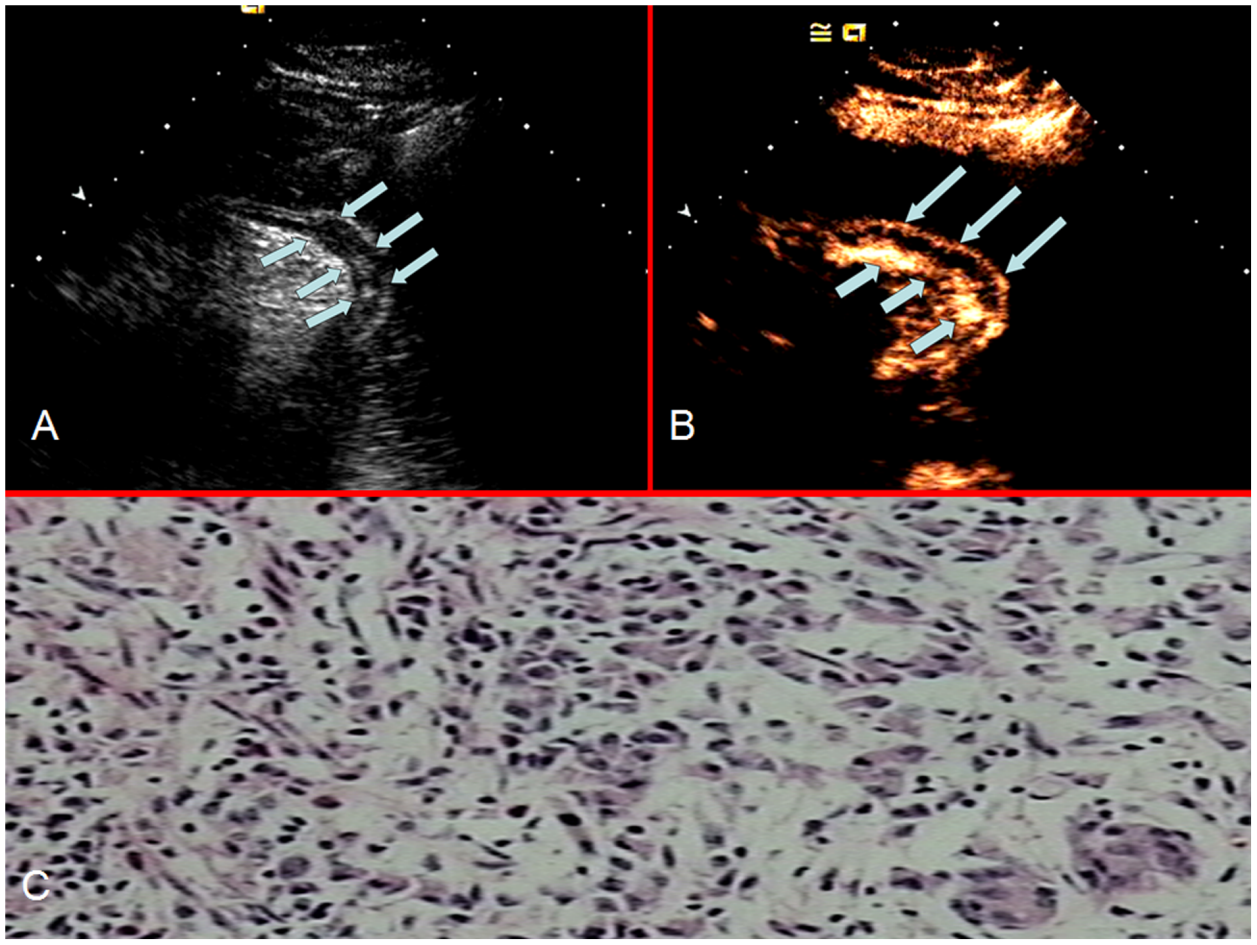
A diffuse thickening of the gastric wall (between the arrows), without a discretely marginated mass or ulceration can be seen on OCEUS (a) and it enhanced with a layered pattern (arrows in a) during the arterial phase using CEUS (b); poorly differentiated adenocarcinoma was confirmed on histopathological examination with H&E staining (x100) (c).

**Figure 3:**
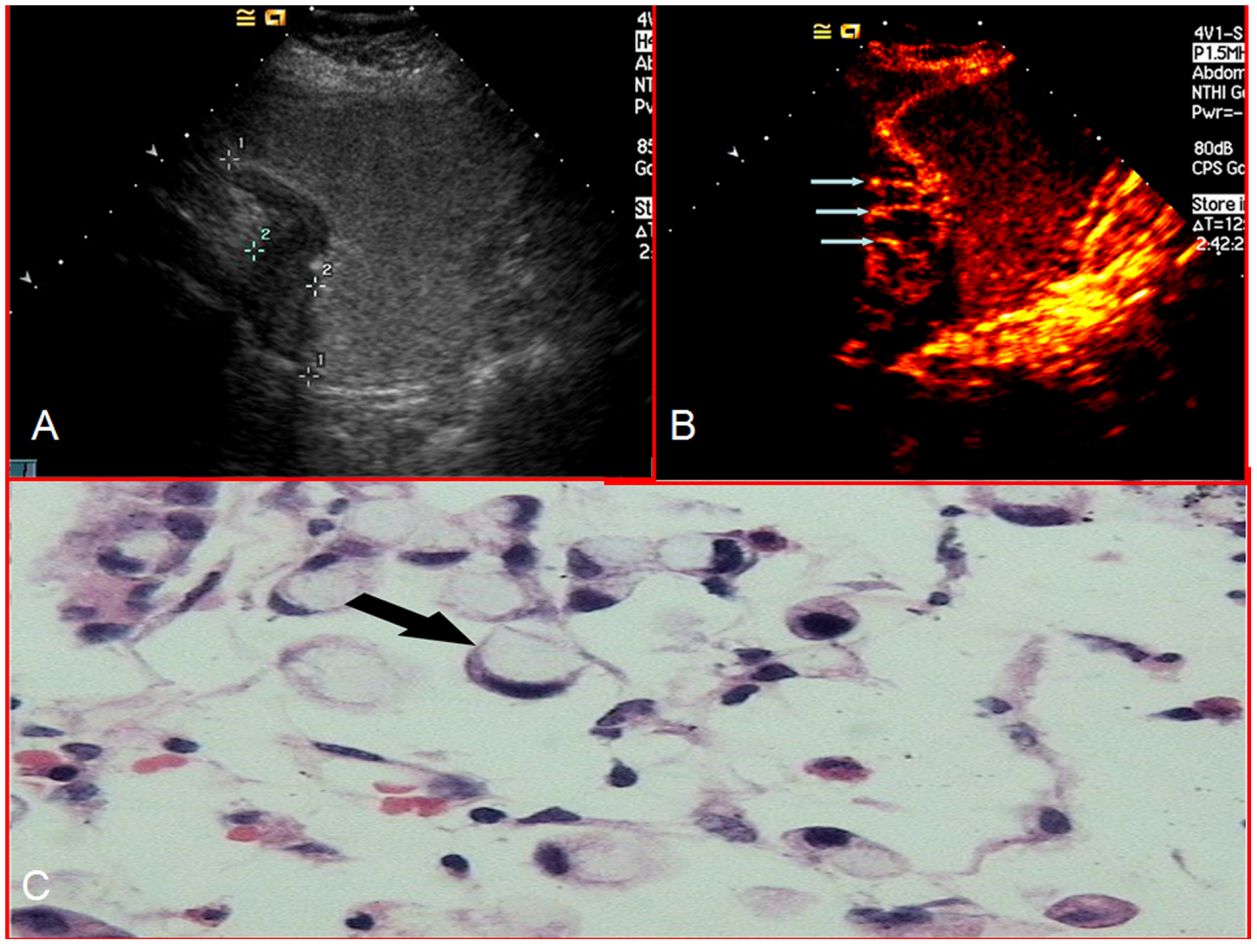
Gastric cancer was demonstrated as a hypoechoic mass (calipers) using oral contrast ultrasonography(a) and enhanced with a striated pattern during the arterial phase using CEUS (arrows in b). Histological findings with H&E staining (x400) showed poorly differentiated adenocarcinoma with signet-ring cells (arrow) (c). STO = Stomach.

The enhancing characteristics of the lesions were observed and the enhancement patterns were recorded and classified as homogeneous or heterogeneous. Homogeneous enhancement was defined as ‘even signal intensity over the whole tumor with no filling defects’ during the early arterial phase (lasting 25 seconds after injection) (Figure 1). Heterogeneous enhancement was defined as ‘uneven signal intensity over the lesion’ during the early arterial phase with layered (Figure 2) or striated (Figure 3) pattern.

Three board-certified abdominal radiologists (SL, YZ and PH, with 12, 13 and 19 years of experience, respectively) who were aware of the presence of gastric cancers reviewed the cine loops of all the 552 lesions without knowledge of other clinicopathological information about the patients. If a disagreement exists, a consensus was reached by discussion. Meanwhile, the interobserver variability of the enhancement patterns was determined by two of same group of radiologists(SL and YZ) who were blinded to each other’s results and to the clinical data. To analyze the intra-observer variability of reading the enhancement patterns, one of the same group of radiologists (SL) evaluated the CEUS video loops at two time-points one month apart and was blinded to the clinical data and to the results of the first evaluation.

The surgically resected specimens were fixed in 85% ethanol and embedded in paraffin. Sections 4 µm thick were cut and stained with hematoxylin and eosin (H&E). The histopathologic findings of the surgical specimens from all patients were retrospectively reviewed by the pathologist (SW, with 23 years of experience), who was unaware of the ultrasound findings. According to the Japanese classification [23], gastric cancers are histologically grouped into five types: papillary adenocarcinoma, tubular adenocarcinoma (including well-differentiated and moderately differentiated types), poorly differentiated adenocarcinoma (including solid or non-solid types), signet-ring cell carcinoma, and mucinous adenocarcinoma. Thus, the differentiated gastric cancers included papillary and tubular adenocarcinomas, whereas the undifferentiated cancers included poorly differentiated adenocarcinomas, signet-ring cell carcinomas, and mucinous adenocarcinomas [23], [24]. Histopathological evaluation of depth of tumor invasion and lymphatic invasion were based on the 6^th^ edition of AJCC TNM staging system [25]. The macroscopic types of the surgical specimens from all of the patients were also classified according to the Borrmann criteria [26].

### Statistical Analysis

SPSS version 13.0 (SPSS Inc. Chicago, USA) was used for the statistical analysis. Continuous variables were described as means and standard deviations (SD). The two-tailed t-Student test was used to assess continuous variables. The chi-square test was used to examine the significance of correlated proportions between the different enhanced patterns groups. Sensitivity and specificity for the heterogeneous enhancement to predict the undifferentiated type of gastric carcinoma were calculated. Additionally, concordance of enhancement patterns scores within and between observers were assessed using Kappa analysis [27] and a good reliability was set as a Kappa-value above 0.75. For all analyses, a *P* value of less than 0.05 was considered to indicate a significant difference.

## Results

The clinicopathological characteristics of 552 patients with gastric cancer are shown in Table 1. The maximum thicknesses of undifferentiated tumors (293 patients) were greater than that of differentiated tumors (259 patients) [(1.63±0.83) cm vs. (1.47±0.66) cm, *P*<0.05], gastric cancers with undifferentiated type had more T3 and T4 depth of invasion than those with differentiated type (71.33% vs. 57.15%, *P*<0.005), more lymphatic invasion (85.67% vs. 71.43%, *P*<0.001), more Borrmann III and IV macroscopic types (57.34% vs. 42.47%, *P*<0.005), and were less likely to receive curative gastrectomy (76.45% vs. 84.17%, *P*<0.05).

**Table 1:** Clinicopathological characteristics of patients with gastric cancer according to differentiation (n = 552).

Variable	Undifferentiated	Differentiated	Chi-square or *t*-value	*P*-value
	(n = 293, %)	(n = 259, %)		
**Sex** (n)			0.062	0.804
Male	175(59.73)	152(58.69)		
Female	118(40.27)	107(41.31)		
**Age** (mean±SD)	65.62±8.79	66.21±13.21	0.624	0.533
**Location of tumor** (n)			0.529	0.913
Lower third	127(43.34)	114(44.02)		
Middle third	93(31.74)	87(33.59)		
Upper third	64(21.84)	51(19.69)		
Whole	9(3.07)	7(2.70)		
**Thickness**, cm (mean±SD)	1.63±0.83	1.47±0.66	2.485	0.013
**Depth of invasion** (n)			14.976	0.002
T1	11(3.75 )	24(9.27)		
T2	73(24.91)	87(33.59)		
T3	142(48.46)	106(40.93)		
T4	67(22.87)	42(16.22)		
**Lymphatic invasion** (n)			16.788	0.000
Absent	42(14.33)	74(28.57)		
Present	251(85.67)	185(71.43)		
**Macroscopic type** (n)			16.128	0.003
EGC	11(3.75 )	24(9.27)		
Borrmann I	31(10.58)	39(15.06)		
Borrmann II	83(28.33)	86(33.20)		
Borrmann III	122(41.64)	83(32.05)		
Borrmann IV	46(15.70)	27(10.42)		
**Curability** (n)			5.134	0.023
Curative	224(76.45)	218(84.17)		
Palliative	69(23.55)	41(15.83)		

**Notes:** Numbers in parentheses are percentages. EGC = early gastric cancer.

The enhancement patterns of the two histological subtypes are summarized in Table 2. Homogeneous enhancement was the more prevalent pattern in differentiated gastric cancers (Figure 1), whereas heterogeneous enhancement was the more common pattern in undifferentiated gastric cancers. Among 293 cases of undifferentiated gastric cancer, 231 cases (78.84%) enhanced heterogeneously (Figures 2 and 3). The remaining 62 cases (21.16%) enhanced homogeneously. Of the 259 cases of differentiated gastric cancer, 188 (72.59%) enhanced homogeneously and 71 (27.41%) enhanced heterogeneously. Chi-square analysis showed that the proportion of heterogeneous enhancement was significantly different between the two subtypes of tumor (Chi-square = 146.735, *P<0*.001). Taking heterogeneous enhancement as the criterion for undifferentiated gastric cancers, the sensitivity and specificity of early heterogeneous enhancement in its diagnosis were 78.84% and 72.59% respectively.

**Table 2:** Enhancement patterns in undifferentiated and differentiated gastric cancer patients (n = 552).

	Undifferentiated	Differentiated	Total
Heterogeneous	**231**	71	302
Homogeneous	62	**188**	250
Total	293	259	552

**Notes:** Sensitivity and specificity were 78.84% and 72.59% respectively for heterogeneous enhancement of gastric lesions to predict the undifferentiated type, Chi- square = 146.735, *P*<0.001.

The enhancement patterns of 552 patients with gastric cancer are shown in Table 3.

**Table 3:** Clinicopathological characteristics of patients with heterogeneous and homogeneous enhancement patterns of gastric cancer (n = 552).

Variable	Heterogeneous	Homogeneous	Chi-square or *t* value	*P*-value
	(n = 302, %)	(n = 250, %)		
**Thickness, cm** (means±SD)	1.50±0.89	1.33±0.84	2.291	0.022
**Infiltration degree** (n)			21.285	0.000
EGC	6(1.99)	29(11.60)		
AGC	296(98.01)	221(88.40)		
**Macroscopic type** (n)			69.951	0.000
EGC, Borrmann I and II	101(33.44)	173(69.20)		
Borrmann III and IV	201(66.56)	77(30.80)		
**Lymphatic invasion**(n)			5.694	0.017
Absent	47(15.56)	59(23.60)		
Present	255(84.44)	191(76.40)		
**Depth of invasion**(n)			9.069	0.028
T1	16(5.30)	19(7.60)		
T2	70(23.18)	82(32.80)		
T3	144(47.68)	104(41.6)		
T4	72(23.84)	45(18.00)		
**Curability** (n)			11.466	0.001
Curative	226(74.83)	216(86.40)		
Palliative	76(25.17)	34(13.60)		

**Notes:** Numbers in parentheses are percentages. EGC = early gastric cancer;

AGC = advanced gastric cancer.

Gastric cancers with heterogeneous enhancement were more often Borrmann III and IV macroscopic types than those with homogeneous enhancement (66.56% vs. 30.80%, *P<*0.001), more commonly T3 and T4 depth of invasion than those with homogeneous enhancement (71.52% vs. 59.60%, *P<*0.05), more often showed lymphatic invasion than those with homogeneous enhancement (84.44% vs. 76.40%, *P<*0.05), and were less likely to receive curative gastrectomy than those with homogeneous enhancement (74.83% vs. 86.40%, *P*<0.005).

The intra- and inter-observer reproducibility were both almost perfect for assessing enhancement patterns of gastric carcinomas with a Kappa value of 0.842 (*P*<0.001) for intra-observer (Table 4) and 0.916 (*P*<0.001) for inter-observer by CEUS (Table 5).

**Table 4:** Concordance of enhancement patterns of gastric cancer by DCEUS according to the findings of the two pairs of observers.

Observers B	Observers A		Total
	Heterogeneous	Homogeneous	
Heterogeneous	**284**	25	309
Homogeneous	18	**225**	243
**Total**	302	250	552

**Notes:** The inter-observer reproducibility was high (K = 0.842, *P*<0.001).

**Table 5:** Concordance of enhancement patterns of gastric cancer by DCEUS according to the findings at 2 separate time intervals of readings.

Second	First		Total
	Heterogeneous	Homogeneous	
Heterogeneous	**296**	17	313
Homogeneous	6	**233**	239
**Total**	302	250	552

**Notes:** The intra-observer reproducibility was almost perfect (K = 0.916, *P*<0.001).

## Discussion

Tumor differentiation is one of the most important prognostic factors for gastric cancer [28]–[30] and there is a general consensus that the histological type should also be considered in selecting therapeutic strategies for these patients [30], [31]. However, the treatment of gastric cancer has become increasingly sophisticated, with therapies tailored to individual cases [32]. Treatment includes a broad spectrum of therapeutic options, from endoscopic mucosal resection for selected mucosal cancers to more radical treatments for advanced cancers. Therefore, accurate preoperative staging, particularly with regard to depth of mural invasion, adjacent organ invasion, nodal involvement, and distant metastases, is vital in determining the most suitable therapy and avoiding inappropriate attempts at curative surgery [4]. Meanwhile, tumor grade refers to the degree of differentiation of the tumor cells and has been shown to correlate with the aggressiveness of the neoplasm [33]. The prognostic impact of histological grading in gastric cancer has been well documented and highlights the poor prognosis of patients with undifferentiated tumors [24].

Our results suggest that the enhancement patterns on CEUS correlated with histopathological differentiation of gastric cancer. Undifferentiated gastric cancers more commonly demonstrated heterogeneous enhancement, whereas differentiated cancers more commonly demonstrated homogeneous enhancement. Tumor neovascularization is associated with de-differentiation and the assessment of tumor perfusion and hemodynamic changes is useful for evaluating the pathological background of the cancer and determining prognosis [34]. Although CT, magnetic resonance imaging (MRI), transabdominal ultrasonography and endoscopic ultrasound (EUS) [35], [36] have been the modalities of choice for preoperative evaluation and staging in patients with gastric carcinoma, the accuracy of each modality for preoperative TNM staging varies, and the agreement between pre-operative imaging staging and post-operative staging by pathology is not perfect, which may affect treatment decisions [37]. Many approaches with imaging techniques, such as CT and double-contrast barium studies of the stomach, have attempted to distinguish between differentiated and undifferentiated gastric cancers [37]–[39]. Park et al. reported that the enhancement pattern on helical CT may assist in distinguishing mucinous from non-mucinous gastric carcinomas [37]. Rossi reported that a 5 to 7 mm hypodense layer and contrast enhancement of the lesion of approximately 80 HU on enhanced CT are suggestive of diffuse gastric cancer, and a poorly enhancing homogeneous thickening of the gastric wall may indicate gastric cancer of the intestinal type [38].

Recently, intravenous contrast-enhanced ultrasound has been used to assess the characteristics of tumors, such as in the differential diagnosis of benign and malignant lesions, tumor angiogenesis, and response to chemotherapy [40]–[42]. Our previous studies [43]–[46] and other reports [47], [48] have shown that CEUS can be used as an accurate, non-invasive, and reliable diagnostic method for detection and preoperative assessment of advanced gastric cancer. The potential advantages of CEUS are absence of ionizing radiation compared with CT and lower invasiveness than endoscopic ultrasound, which also carries the potential risks of perforation, pancreatitis, bleeding, and infection.

Though different enhancement patterns could be distinguished in differentiated and undifferentiated cancers in our series, there was considerable overlap. Among 293 undifferentiated cancers, 62 showed homogeneous enhancement, while 71 differentiated cancers had heterogeneous enhancement patterns. The sensitivity and specificity of heterogeneous enhancement of CEUS in diagnosing undifferentiated gastric cancer were 78.84% and 72.59% respectively. Our previous study also implied that the heterogeneous pattern was more common in diffuse than in intestinal cancers using the Lauren classification [45].

Intestinal and diffuse gastric cancers differ pathologically in cellular cohesion [37], and this could be the main reason for the different enhancement patterns on CEUS. In diffuse gastric cancer, clusters of tumor cells infiltrate the layers of the stomach wall; therefore, desmoplastic reaction and inflammatory peritumoral reaction are limited to the gastric wall, and the parietal surface is often smooth and regular, while in intestinal gastric cancers, cells are more closely linked and organized in solid or glandular structures that completely replace the gastric layers. Although the Lauren and histopathological classifications differ somewhat, according to Lauren’s classification, intestinal and diffuse gastric cancers correspond to the differentiated and undifferentiated types of gastric cancers used in the present study [49].

Our findings are supported by previous studies, which have shown that the distribution of vessels is heterogeneous and tangled in heterogeneously enhanced tumor tissue, and orderly in homogeneously enhanced tumors [50], [51], and this correlates with histological differentiation on pathological examination [38]. Interestingly, our study showed that the enhancement patterns on CEUS not only correlated with the histological differentiation of gastric cancer, but also with the macroscopic type, depth of invasion and lymphatic involvement. Patients with heterogeneous enhancement in their gastric cancers were more often Borrmann III and IV macroscopic types, were more commonly T3 and T4 in thickness and more commonly showed lymphatic invasion. Shin et al. reported similar results using dynamic CT [52]. Additionally, those with a heterogeneous enhancement pattern were less likely to have curative gastrectomy. Our results suggest that patients whose gastric cancer had heterogeneous enhancement have a poor prognosis. The intra- and inter-observer agreements on the enhancement patterns of CEUS showed Kappa value of 0.916 and 0.842 respectively demonstrating good reproducibility.

Our study has some limitations. First, the data analysis was retrospective and only included patients referred to our hospital for surgery. Although blinded to the endoscopic and CT findings, and to the surgical and histopathologic results, the observers were aware of the presence of a gastric tumor. Second, we did not analyze the survival of the patients with different enhancement patterns on CEUS and this is the subject of ongoing research. Third, we only analyzed qualitative enhancement patterns on CEUS. Fourth, for historical reasons, we used the 6^th^ edition of the TNM staging system, which was somewhat different to the recently published 7^th^ edition [53].

In conclusion, CEUS is unlikely to challenge the role of optical endoscopy because CEUS cannot make a pathological diagnosis of gastric cancer. However, as enhancement patterns of CEUS correlated with the clinicopathological findings of gastric cancer, CEUS may improve planning the treatment of gastric cancer.
